# The Stressor in Adolescence of Menstruation: Coping Strategies, Emotional Stress & Impacts on School Absences among Young Women in Nepal

**DOI:** 10.3390/ijerph18178894

**Published:** 2021-08-24

**Authors:** Siobhan K. Yilmaz, Alok K. Bohara, Swati Thapa

**Affiliations:** 1 College of Population Health, University of New Mexico, Albuquerque, NM 87131, USA; 2 Department of Economics, University of New Mexico, Albuquerque, NM 87131, USA; bohara@unm.edu; 3 Lumbini Center for Sustainability, Pratiman-Neema Memorial Foundation (PNMF), Siddharthanagar 071-522505, Nepal; swati_thapa@hotmail.com

**Keywords:** conditional mixed-process (CMP), psychological wellbeing, school support, cultural support, primary data, menstruation, hygiene, principle component analysis (PCA), MCA, stress and coping

## Abstract

Throughout the developing world, girls face hardships surrounding menstruation, often resulting in poor emotional wellbeing and missing school. Providing ways to keep girls in school will increase their educational and earning potentials, which will ultimately trickle down to improving the economic standing of nations in the next generation. Informed by the Transactional Model of Stress and Coping, this work evaluates the roles that cultural and school environments play in appraisals of menstruation as a major life stressor for adolescent females and the impacts of emotional stress on missing school. Using primary survey data from schools in Nepal, robust results are found to support the theoretical framework based on conditional mixed-process (CMP) estimation with fixed effects, utilizing multiple index building techniques. Strong cultural norms during menstruation appear to increase the probability of girls self-reporting emotional stress, while the presence of hygiene supporting infrastructure at schools reduces this outcome. Furthermore, there is strong support for the finding that the presence of emotional stress during menstruation increases the likelihood of not only missing school but also for an extended period of time. Our findings motivate increasing government policies to provide stronger hygiene infrastructure in schools to improve successful coping skills and attendance rates.

## 1. Introduction

Research on water, sanitation, and hygiene (WASH) in developing nations include a rapidly growing focus on menstrual hygiene management (MHM) [1]. The confusion/surprise many females face when confronted with the onset of menses is often attributed to a lack of knowledge and proper facilities [2,3,4,5,6,7]. Such outcomes are exacerbated by cultural taboos/stigmas still currently associated with menstruation in many developing countries [8,9,10]. South Asian countries, in particular, have been noted for the depth of restrictions surrounding limitations in types of food, rules on which cultural/religious events menstruating females may attend, and who they may interact with/near [11]. Such restrictions are deeply rooted in the traditional beliefs and teaching of Hindu culture, which possess strong superstitions surrounding the impurity of blood [12].

In Nepal, a predominantly Hindu nation, such taboos can be severe. This is most strongly exemplified through the practice known as *Chhaupadi*, wherein females must live in separate huts while menstruating [13,14]. While officially banned in 2005 by the Nepali government, one still finds it practiced, especially rurally [15]. With even less access to properly wash and dry menstrual hygiene products, just as in other nations with strong isolation practices, many women/girls end up reusing soiled products or resorting to old rags or even leaves [16,17]. Women, thus, still face the likelihood of such reproductive health problems as abnormal discharge, itching, pain/foul-smelling menstruation, and burning urination (e.g., UTI) from improper care during isolation times [18,19]. Furthermore, multiple incidents of death associated with menstrual isolation have been documented, even among literate women who are engaged in helping women improve their reproductive health [20]. Additionally, there has been evidence of intimate partner and sexual violence inflicted upon women during their time of isolation [21]. Altogether, these social constraints/stigmas establish an environment in which lack of knowledge and lack of supplies only compound the level of stress associated with menstruation for young women, leading to potential long-term negative outcomes.

Among the most important socio-behavioral consequence to consider is that of missed opportunities at school, for which the World Health Organization (WHO) emphasizes the need to focus on better school facilities. An accumulation of missed school days may ultimately lead to girls dropping out of school altogether [6,22,23,24,25]. This trend opens the door for such practices as child brides and female genital mutilation–practices that international initiatives such as the Millennium and Sustainable Development Goals [26,27] seek to eradicate. In addition, by keeping girls in school, their long-term educational and thus earning potentials are higher. A higher-earning population translates to an overall better economic standing for the nation. Progress towards these goals will be enhanced through strong empirical evidence which justifies further research and presents potential policy approaches.

Work, both in Nepal and globally, focused on MHM has been based primarily on qualitative or mixed-methods studies, typically capturing information on the lack of knowledge, specific interventions, and attendance rates, with thematic associations to psychosocial environmental factors [28]. The growing body of purely quantitative-focused work in the field has primarily focused on the impacts of infrastructure or education-based interventions and their impacts on school absences, with mixed results [29,30,31,32,33,34,35,36]. While this strand of more quantitative-focused research analyzing MHM is growing, one should note a gap in its attention to the cognitive/emotional experiences during menstruation.

So far, attention to cognitive components often only appears in quantitative studies as a single question revealing such elements as shame/fear or lack of confidence [25,37,38]. There are also studies indicating improvements in psychological factors (e.g., depression/stress/anxiety) from hygiene education interventions [39,40], but lacking strong quantitative empirical analysis. Theory-based literature on menstruation is much stronger, where among other things, one finds studies discussing the emotional content surrounding menstrual shame which reinforces gender inequalities [41], and the role of objectification theory in emphasizing how females adopt the sexualization of women that society creates and internalize it, leading to views of menstruation as “bad” [42,43].

Researchers involved in the global discussion of MHM think that now is the time for the determination of which factors will best enhance females’ experience during menstruation [5,44,45], including improving awareness of the mental health issues women face, along with mediating and protective factors [46]. If we can begin to tackle all of these elements simultaneously, this field of research will have much more impact and greater gains can be made to ensure that young girls can remain in school and become economically productive members of society. In that vein, this work, founded in the Transactional Model of Stress and Coping incorporates the emotional elements of managing menstruation into existing quantitative findings regarding missing school, by establishing a specific pathway that is empirically evaluated using multiple estimation techniques of a two-equation system.

## 2. Materials and Methods

### 2.1. Conceptional Framework

Stress has been known to contribute to illness both through direct physiological effects (e.g., hormone fluctuations, flight-or-fight responses [47]) and indirect effects via maladaptive health behaviors. However, the ways individuals cope with stress ultimately influence psychological and physical health outcomes. Reactions can promote or hinder healthful practices, and influence motivations to engage in activities that promote health/wellbeing, including motivations/abilities to attend school.

One modeling framework looking at stressful life events is the Transactional Model of Stress and Coping [48,49], which positions stressful life events as person-environment interactions. Under this framework, the impact of an external stressor is mediated by a person’s appraisal of the stressor itself and the psychological, social, and cultural resources at her disposal to aid in dealing with the stressor. When that stressor is appraised as having a major impact greater anxiety and situation-specific distress results [50]. Such distress is exacerbated if appraisals show a lack of resources/controllability [51,52].

**Figure 1:**
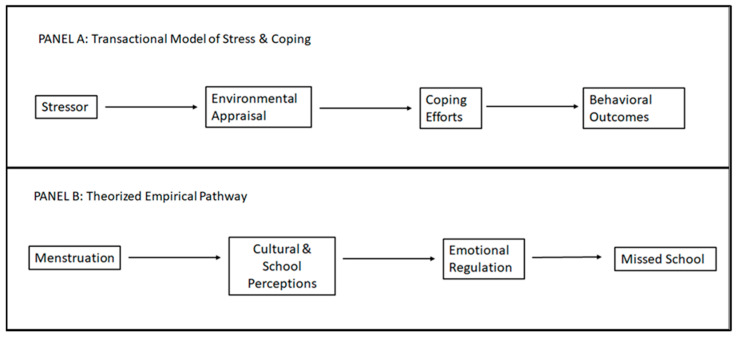
Conceptual framework.

As shown in Panel A of Figure 1, the success of coping efforts, based on environmental appraisals, is often measured through specific health behaviors or emotional well-being. Menstruation for adolescent Nepali females has been shown to limit their ability to access school and other social/cultural events. However, stronger analysis of the contributing factors and associations between various factors is needed, and that is where this model may serve as a useful guide.

Work specifically from Nepal has presented thematic findings linking such elements as lack of knowledge, culture/tradition, and environmental constraints with stress and coping issues, along with such outcomes as reduced school attendance [24,53,54]. Additionally, a meta-synthesis of multiple (primarily) qualitative studies of menstruation experiences across the developing world [28] developed an integrated model linking the socio-cultural context and resource limitations girls/women possess to their experience of menstruation and potential subsequent impacts on such elements as psychological/physical health and education.

Thus, for this work, we take some of these larger ideas from the qualitative literature and apply the specific pathways from the Transactional Model of Stress and Coping (shown in Panel B), wherein the key appraisals are those of the cultural and school environments, in evaluating one’s ability to deal with the stressor of menstruation. Coping efforts (e.g., emotional outcomes) which come out of such appraisals, we believe, then have the additional impacts on such behavioral outcomes as missing school.

### 2.2. Empirical Specification

Given the hypothesized pathway presented above and informed by the Transactional Model of Stress and Coping, we believe that in the face of menstruation, the appraisals young women have of their supporting environments will influence how well they cope. The success of such coping can be captured in measures of emotional stress, which we believe in turn will impact their abilities/willingness to attend school. To represent this system, set out below are the two empirical equations for estimating our key outcomes of emotional stress and school absences:

(1) $$Em_{i}^{*}=\alpha_{1}Env_{i}+\alpha_{2}X_{i}+u_{1i}$$

(2) $$Sch_{i}^{*}=\beta_{1}Em_{i}+\beta_{2}X_{i}+u_{2i}$$

$Em_{i}^{*}$ and $Sch_{i}^{*}$ are two latent variables (qualitative) representing Emotional Stress and School Absences. Given our lack of a complete instrument to measure emotional stress, $Em_{i}^{*}$ is proxy represented by a self-reported measure of feeling sad/lonely during menstruation. $Env_{i}$ is a vector of three environmental variables {*SchEnv, CommEnv, FamEnv*}. *SchEnv* is an index representing appraisals of the school environment (physical infrastructure and hygiene education). *CommEnv* and *FamEnv* are indices representing appraisals of the community and family cultural environments, respectively. The vector *X_i_* contains socioeconomic and demographic controls including age, caste, and school-level fixed effects. In this two-equation model, we allow for non-zero covariance, cov ($u_{1i},u_{2i}$) ≠ 0, to enable simultaneity between the emotion and the schooling outcome equations.

### 2.3. Hypotheses

Recall that appraisal of a stressful event involves perceptions and awareness of supportive elements to effectively cope. When females face menstruation, they assess whether there is supporting infrastructure (e.g., hygiene materials) and/or cultural support/knowledge to aid in coping. Findings show that parents fear sending their girls to school, and girls fear judgment from others [55] for not having underwear/leaking. The presence of the right infrastructure could reduce such fears and allow for better coping. The sense of controllability and self-efficacy would improve, leading to more effective coping strategies. Thus, our first hypothesis is:

**Hypothesis** **1** **(H1).** The presence of menstruation-supporting infrastructure and education in schools (SchEnv_i_) will help adolescent females feel less emotional stress $\left(Em_{i}^{*}\right)$.

Evidence has also shown that social support can be a “stress buffer” [56], but perceptions of lack of social support can remove any potential buffering benefits. The practice of isolating menstruating females [18,57], as well as the noted lack of knowledge about menstruation in Nepal [2,3,58], appears reminiscent of avoidance and denial strategies, generally seen as maladaptive [59,60].

There is also evidence that when key social supports actively discourage the disclosure of feelings about a stressor, this ultimately leads to adverse psychosocial outcomes [61,62]. In cultures with a strong subordination of women, women are often found to be at greater risk of developing a number of mental disorders [63,64,65], including the “self-silencing” cultural expectation among women in Nepal [66]. Thus, existing evidence appears consistent with our conceptual framework and leads to our second hypothesis:

**Hypothesis** **2** **(H2).** Strong cultural norms which restrict adolescent girls during menstruation (CommEnv_i_, FamEnv_i_) will lead to more emotional stress $\left(Em_{i}^{*}\right)$.

One potential manifestation of avoidant coping is skipping school. Better coping practices from improved school infrastructure/environment and better social support we expect would be reflected in better emotional wellbeing. This in turn is likely to reduce emotional barriers, allowing girls to more easily attend school during menstruation. Thus, our final hypothesis is as follows:

**Hypothesis** **3** **(H3).** Presence of emotional stress $\left(Em_{i}^{*}\right)$. during menstruation will increase the likelihood of girls missing school $\left(Sch_{i}^{*}\right)$.

### 2.4. Data & Survey Design

Data for this study comes from two regions in south-central Nepal, allowing us to capture two different localities. Siddarthnagar, in the central Terai (plains) region of the country, has a major zonal 135-bed hospital located within a half-hour of driving distance from the city. The area also boasts two medical schools, one eye hospital, one agriculture college, an engineering college, one science college, and several two- to four-year colleges. As such, this area provides a reasonable picture of an active urban area (outside Kathmandu) where education is valued and access to modern technology is more viable. Purkot, in the Hills region of Nepal, is recognized as a slightly more rural area with comparatively lower literacy rates and access to modern technology, as well as, higher proportions of citizens belonging to “lower” castes.

Our primary survey data was collected in conjunction with a non-profit organization that provided female hygiene packets to Nepali females in May 2016 (Siddarthanagar) and December 2017 (Purkot). Along with providing these reusable kits, good for up to three years, educational information about female health and hygiene was provided. Plus, a self-administered, paper-and-pencil survey was administered to females at a school in each region, where confidentiality was assured to enable respondents to be more forthcoming in their answers on this sensitive topic. (Informed consent was sought in the administration of the survey and was approved by local authorities. Parents and teachers were present at this event and representatives of the Pratiman-Neema College were on hand to ensure that the study was carried out in a manner consistent with the ethical standards of the Declaration of Helsinki). Two schools were surveyed to allow for variation in data and to prevent school bias. We focused on those grade levels placing girls above the age of menstruation initiation, and the supply of menstrual hygiene kits set our limit on maximum sample size.

Survey questions were asked regarding basic demographic information (i.e., age, religion, caste, family possession of cement home/land), along with information on current knowledge and practices regarding menstrual hygiene (i.e., genital cleaning, hand washing, pain treatments). In addition, of importance for this study, the survey also included information on current school infrastructure (i.e., presence of trash bins, emergency menstrual hygiene kits, and soap for washing hands) and cultural restrictions women face in their home environment during menstruation (i.e., separate sleeping quarters, not allowed in the kitchen, etc.). Key to this work was the inclusion of a question evaluating whether the girls felt sad or lonely during their menstruation period and assessments of whether or not the respondents have had to miss school due to their menstruation (along with a range of how many days were missed the previous month).

### 2.5. Key Variables

The dependent variable from Equation (1), Emotional Stress ($Em_{i}^{*}$), is a binary (1/0) indicator of feeling sad/lonely during menstruation (Survey Question: “Do you feel lonely and sad during your menstruation cycle?”). There are two representations of the dependent variable from Equation (2), School Absences ($Sch_{i}^{*})$. The first specification (Specification A) is a binary (1/0) indicator of having missed school due to menstruation (Survey Question: “How many days in the last month have you missed school due to your menstruation?”). The second (Specification B) has ordinal coding for numbers of days missed the prior month (zero, 1–2 days, and 3+) (Survey Question: “How many days in the last month have you missed school due to your menstruation?”). Our key explanatory variables from the column vector $Env_{i}$ in Equation (1) represent the appraisals of respondents’ environments. Principle component analysis (PCA) was used to build these indices, with findings confirmed using multiple correspondence analysis (MCA). (PCA is a standard statistical technique for data reduction. The goal is to find unit-length linear combinations, where the first principle component has the maximal overall variance, and each additional principle component has the maximal variance among all unit length linear combinations that are uncorrelated to the first component [67]. MCA is a generalization of PCA, where the variables to be analyzed are categorical (i.e., binary) not continuous. MCA analyzes the inter-individual variability, trying to extract which dimensions separate extremely different individuals from average individuals. Additionally, there is an assessment of the links between variables, and which categories of one variable are connected to categories of another [68]). The variable representative of awareness of school support is based on questions pertaining to the reported presence/absence of certain key hygiene facilities/infrastructure at school (e.g., sanitation kits, toilet, soap, disposal bins, hygiene education). Those variables capturing the cultural environment in which girls live at home assess both more family-based elements (e.g., forced isolation, ability to meet with family) and more community-based elements (e.g., permission to enter worship room, cultural functions, and kitchen). (Survey questions and their coding are available in Table S4 in the Supplementary Materials). Entering each of these questions individually into our empirical regressions would eat up too much power. Additionally, the use of PCA allows us to better capture the multifaceted nature of both the school and cultural environments in question for this study, where usage of a single proxy variable would seem insufficient.

In terms of control variables, *Age* is included as it would be expected that management of menstruation may change over time. Coping efforts may become more fully honed the longer girls have had to manage menstruation [69], and appraisals of support may shift. Descriptive summary statistics of all key and control variables can be found in Table 1.

### 2.6. Estimation Strategies

#### 2.6.1. Single Equation Estimation

The first stage (e.g., baseline) of empirical estimation was through separate regressions of each outcome equation. Probit estimation was used for single equation estimation of Equation (1) and Specification A (e.g., binary) of Equation (2). Ordered probit was used for Specification B (e.g., ordinal) of Equation (2).

Estimates presented in this paper used school fixed effects and caste indicators. Additional control variables considered were current hygiene product use (base as old rags/cloths), marriage status, and a wealth index. Presentation of model fitting for these single equations is found in the Tables S1–S3. Table 2 presents the results from a summary of best-fit estimates based on the separate equation analysis.

Due to the possibility of a sort of self-selection into the survey, in that these girls are not a random nor necessarily representative sample of all teenage girls in Nepal, we also ran our single equation estimations using bootstrapped errors. Additional robustness checks included running the most preferred estimations a sample including older females (ages 21–44) also surveyed. We also checked our estimations (in all specifications) with the inclusion of a variable representing reporting of a teacher to discuss worries with.

#### 2.6.2. Simultaneous Estimation of Emotional Stress & School Absences

Results of single-equation estimates could be biased due to unobserved characteristics that determine both emotional stress and school attendance, which would result in endogeneity concerns from the correlation between the error terms of the two outcome equations [70]. While instrumental variables (IV) is a common technique for dealing with such concerns, binary choice models are almost exclusively estimated via maximum likelihood estimation (MLE), as we have done.

In analyzing Equations (1) and (2), where both outcomes ($\;Em_{i}^{*},Sch_{i}^{*})$ are represented by binary indicators (Specification A), we used bivariate probit estimation. This approach allows one to consider non-zero covariance between the two estimation equations. We present raw estimates with only fixed-effects (Model 1) and a second where we also include caste indicators (Model 2), found in Table 3. The marginal effects of these estimates are presented in Table 4, given limited meaningful interpretation from raw coefficients of probit estimation.

In this study, we also have the unique opportunity to represent our school absence variable with an ordinal structure. Simultaneous estimation of Equation (1) and Specification B of Equation (2) presents the situation of two separate coding structures for the two outcome variables. Bivariate probit cannot be used to estimate that simultaneous equation system. Therefore, we implemented conditioned mixed-process modeling (CMP), (CMP Modeling is based on the premise that because the normal distribution has a natural multidimensional generalization, models can be combined into equation systems where errors share the multivariate normal distribution. Thus, one is capable of estimating a system such as a bivariate probit, where both outcomes are binary (latent) variables, or one can estimate a “mixed”-model system with one binary outcome and one ordinal, and allow for non-zero covariance between the error terms [71]) and present the raw results and marginal effects in Table 5 and Table 6.

## 3. Results

### 3.1. Basic Statistics

The average age of our sample (*n* = 281) was 16.6 years old (SD = 1.96). In terms of products used during menstruation, 22.8% use old rags/clothes, 66.9% use disposable pads, and 10.3% reusable products. In total, 60.1% of women claimed that they experience extreme pain during their menses, and yet, only 13.3% report using a hot pack, 21.4% using pain medicine, and 21.3% going to a doctor to deal with their menstruation discomfort. 89.5% of women report washing hygiene supplies with just soap, with only 8.7% using some form of antiseptic. Almost all report that they wash their hands after changing hygiene products, but less wash their hands prior. Overall, it appears that these girls, on average, practice some key hygiene behaviors, but the presence of proper infrastructure is important to maintaining these healthy behaviors.

As mentioned, one of the biggest concerns of improper MHM is the consequence of lost days of school which limits girls’ eventual economic success. Of all girls sampled, 42.1% reported knowing someone who had to drop out of school due to menstrual problems. Overall, when asked how hard it was for them to manage work and/or school during menses, 68.4% of those surveyed claimed it was hard or very hard. So, in the face of 32.7% reporting that they miss school due to menstruation, and 48% of our sample reporting that they experience emotional stress during menstruation, there is still a need for improvement in how menstruation is dealt with among these Nepalese female students.

### 3.2. Single Equation Estimation Results

We ran three single-equation estimations using probit for Equation (1) and Specification A of Equation (2) and ordered probit for Specification B of Equation (2), under various control situations (see Tables S1–S3). Table 2 presents a summary of the best-fit of each single equation estimate based on the minimum Akaike information criterion (AIC) (Akaike information criterion (AIC) deals with the trade-off between the goodness of fit of the model and the simplicity of the model [72], where the lowest AIC figure among a set of potential models is deemed the most useful).

In column 1 are the results from estimating the impacts of environmental assessments of support ($Env_{i})$ on emotional stress ($Em_{i}^{*})$. The appraisals of the school environment (*SchEnv_i_*) appear to be significant (−0.299, *t* = −3.15), indicating that more menstruation supporting infrastructure at school reduces the likelihood of a girl reporting feeling sad/lonely during menstruation. Column 2 reports the impacts of emotional stress ($Em_{i}^{*})$ during menstruation on missing school $\left(Sch_{i}^{*}\right)$, showing a significant positive effect (0.58, *z* = 3.42). Significant results are also found for the impact of emotional stress ($Em_{i}^{*})$ on days of school missed $\left(Sch_{i}^{*}\right)$ in Column 3 (0.341, *z* = 2.12). In both cases, results indicate that having emotional stress during menstruation increases the chances of missing school (and missing more days) during menstruation.

### 3.3. Simultaneous Estimation Results

#### 3.3.1. Specification A: Binary-Binary Dependent Variables

With concerns over the endogeneity of emotional stress in our school absence equation, we undertook estimation of the two outcome equations simultaneously, allowing for covariance in their error structures. Presented in Table 3 are the parameter estimates from bivariate probit estimation of the equation system, where $Sch_{i}^{*}$ is binary (Specification A).

Under this approach, we find significance under both models for all three representations of appraisals of environmental support, with *CommEnv_i_* and *FamEnv_i_* increasing the likelihood of emotional stress (0.146, *z* = 2.57; 0.148, *z* = 2.54, respectively) and *SchEnv_i_* decreasing this likelihood during menstruation (−0.25, *z* = −3.05). Rho (ρ), representative of the associations between the two equations indicates a significant correlation (*z* = −5.95) between the errors of the two equations, supporting the choice to simultaneously estimate them. As shown in Table 4, the average marginal effects of such results indicate that awareness of supporting infrastructure at school reduces the likelihood of emotional stress by 8.1–9.2% (*z =* −3.19). Alternatively, appraisals of strong restrictions in both the family and community environments are estimated to increase the likelihood of emotional stress by around 5% (*z* = 2.63).

We also see strong significant results of the impact of emotional stress ($Em_{i}^{*})$ on school absences $\left(Sch_{i}^{*}\right)$ during menstruation. Table 3 shows that there is a strong positive association between the presence of emotional stress and missing school (regardless of controlling for caste) (1.82, *z* = 15.1). The marginal effect (Table 4) indicates that the likelihood of missing school increases 47.4–48.9% (*z* = 36.7) if the respondent also reports feeling sad/lonely during menstruation.

#### 3.3.2. Specification B: Binary-Ordinal Dependent Variables

Results of simultaneous estimation of Equation (1) and Specification B of Equation (2) are found in Table 5 and Table 6. The parameter estimates of environmental support appraisals under this estimation indicate that awareness of menstruation supporting infrastructure at school reduces the likelihood of emotional stress $\left(Em_{i}^{*}\right)$(−0.226, *z* = −3.08), while restrictions in the family environment increase this likelihood (0.15, *z* = 2.45). These findings are robust to the inclusion of caste controls.

Marginal effects (Table 6) indicate that awareness of more support for menstruation at school reduces the likelihood of reporting emotional stress by 7.7–8.4% (*z* = −3.18), while stronger family controls increase this likelihood by around 5.7% (*z =* 2.5).

Parameter estimates indicate a positive association between the presence of emotional stress and missing more days of school due to menstruation in the last month (1.67, *z* = 11.76). Again, this strong significance is unaffected by the inclusion of caste dummies. Presentation of marginal effects (Table 6) reveals this association to be stronger for more reported days missed. While the presence of emotional stress increases the chances of missing 1–2 days by 14.2–15.1% (*z* = 3.51), it increases the chances of missing 3 or more days by 31% (*z =* 5.18). While counterintuitive, the negative marginal effect of emotional stress on the first-level outcome of days missed indicates that the presence of emotional stress during menstruation reduces the likelihood of missing zero days of school by 45–46% (*z =* −19.26), consistent with the marginal effects found in Table 4. Rho again indicates a strong correlation between the equations.

#### 3.3.3. Robustness Checks

Checking single equation estimates with bootstrapped errors showed no change in sign and minimal loss in significance. Inclusion of a dummy for the perceived presence of a teacher with whom the girl could share her problems/concerns also did not impact significance/sign, nor add any additional useful explanatory power. Examination of impacts on our results from including a larger estimation sample of women (likely teachers) aged up to 44 produced no change in sign or significance of our key results. Nor did it change any of our overarching conclusions. Results of these analyses are available upon request.

## 4. Discussion

Literature provides evidence that knowledge is powerful in changing behavior and can be done through schools [33,36,73]. Given that cultural norms or taboos are very hard to change with policy and our strongest results refer to the mitigating power of school infrastructure (Hypothesis 1), the implication is to focus policy at schools in future MHM initiatives. With greater awareness of supporting infrastructure, girls would then have better coping skills, resulting in improvements to their psychological well-being, which can have further additional benefits on improved attendance rates. However, such initiatives must be carried out with a wide range of considerations, allowing for comprehensive support, as evidence has shown that unidirectional and sole-medium interventions are often ineffective [74,75,76]. Such a proposition would be consistent with the evidence of a recent multi-faceted intervention in Africa showing that girls reported decreased anxiety about their next period improved comfort managing menstruation with qualitative data indicating potential improvements in menstrual-related school absenteeism [40]. This intervention included such elements as training teachers to improve hygiene education, training in the use of the menstrual kit, and improvements to school WASH facilities.

Of additional consideration is Snel and Shordt’s [77] argument that children can be change-makers. Not only do initiatives to improve school learning environments allow students to be healthier, but they also allow for the dissemination of hygiene information which can be taken home and shared with other family members. By targeting younger school-aged females, efforts may be able to affect behavior beyond the classroom environment. There is preliminary evidence of this phenomenon from India, showing that older generations of women are beginning to indicate desires for their daughters to be less influenced by superstitions [13,78].

We do acknowledge some of the limitations of our study. One is that all of our variables are self-reported. Self-perceived notions of loneliness are likely to differ across individuals and awareness of the presence/absence of the various environmental support system variables, which form the indices, may differ across contexts. Plus, we recognize that there may be other elements not specifically measured which may additionally capture the social norms around menstruation, stigma, and access to menstrual materials [79], but our use of PCA we believe better captures the constructs under examination better than a single proxy element. Further, the answers are given to those questions on the school (e.g., presence of a bin, emergency materials, and soap) and cultural (e.g., degree of isolation/cultural restrictions) environments are a reflection of the different awareness levels of individuals. It is such awareness that forms the basis for the conceptual framework which underlies this study. Each person’s awareness and observations of the supporting environment and its potential ability to aid in coping with a major life stressor determines how well she ultimately copes.

We note that this work is a cross-sectional snapshot and the survey instruments are drawn mostly from published literature. We also have not included certain extensions to the Transactional Model of Stress and Coping which have been used by others, including coping styles, optimism, overall positive psychology, or accounting for individuals as being “information seekers” versus “blunters” [80]. Further, we note that given the small number of clusters (e.g., three), we opted to use fixed-effects to control for school heterogeneity rather than using clustered standard errors. Additionally, we again acknowledge that the use of the self-reported feeling of being sad/lonely is only a proxy representation of the concept of emotional stress.

There is also some research on emotions and behaviors during menstruation which might indicate a slight bias of our findings. A meta-analysis of menstrual cycle effects on mental health outcomes, due to hormonal fluctuations, found that there is a greater risk of suicide deaths/attempts and greater risk of psychiatric admissions during menstruation [81]. Researchers have also found that self-esteem was lowest and paranoid thinking highest in the para-menstrual period (3 days before and after menstrual flow) as compared to mid-cycle [82]. However, those findings are based on data collected in the developed world and may not be as relevant to our sample. While it would be comforting to be able to account for these elements which might bias the data, our data does not include the menstrual cycle stage of girls at the time of surveying. However, it is reasonable to assume that not every girl would be at the peak hormonal period when surveyed, meaning analysis of averages is likely to wash out some of this potential bias. Together with more clearly specifying a psychological outcome based on diagnostic criteria of the psychological state being measured (e.g., depression, sadness, anxiety, loneliness), the implications of this work could be more fully fleshed out.

Finally, as older women and those girls who have already dropped out may not be able to benefit directly from policies aimed at improving school infrastructure, future research could benefit from further examination of means to help them combat their loneliness and improve their coping skills, likely through more focus on improving social support. Perhaps such changes may be possible through the aforementioned link of children as change-makers.

## 5. Conclusions

As MHM becomes an important initiative in the developing world, there is a call for stronger empirical and quantitative analyses. Appealing to the Transactional Model of Stress and Coping framework, we examined the impact on Nepali females of coping efforts on self-reported emotional wellbeing during menstruation and associated impacts on school attendance. Results of several estimation techniques show that the cultural environment girls face increases their probability of emotional stress (e.g., feeling sad/lonely), while the presence of school infrastructure to support menstrual hygiene reduces this probability. In turn, the presence of emotional stress increases the likelihood of missing school (and more days) during menstruation. We also find evidence of the school environment having a statistically significant indirect effect on the likelihood of whether or not one misses school, through the emotional stress variable.

There is thus, strong support for both our first and third hypotheses and modest support for our second hypothesis, across all estimation approaches. While we note that our empirical results cannot directly speak to the clinical significance of the size of effects, we are very intrigued by the results captured. Our work supports the huge role that MHM in schools can play in improving the ability of young women to cope with menstruation [83], and future multi-faceted interventions should leverage the access and influence provided therein. Improvements in coping support will aid in improving the emotional state of young women, which will further encourage girls to remain in school so that they can become more useful and influential in the future growth of their society.

## Figures and Tables

**Table 1:** Descriptive statistics of variables.

Variables	Description	Mean (S.D.)	Percentage	Min/Max
OUTCOME VARIABLES				
Emotional Stress	=1 if female self-reports as feeling sad or lonely during menstruation, 0 otherwise		48%	0/1
Missed School	=1 if female self-reports missing school due to menstruation, 0 otherwise		32.7	0/1
Day Miss School	=1 if missed 0 days of school last month due to menstruation, 2 if missed 1–2 days, 3 if missed 3+	1.331 (0.554)		1/3
EXPLANATORY VARIABLES				
School Environment	PCA index of (1/0) self-reported yes answers to the existence of sanitation supplies, separate toilet, soap, disposal bin, & hygiene education at school	−1.39–08 (1.45)		−2.16/2.67
Community Cultural Environment	PCA index heavily loaded with (1/0) self-reported yes answers to not being able to enter prayer room, participate in cultural functions, or enter the kitchen	5.73–09 (1.25)		−4.51/1.90
Family Cultural Environment	PCA index heavily loaded with (1/0) self-reported yes answers to not being able to stay in the same house & not meet family	5.08–09 (1.04)		−0.789/5.44
Age	Age	16.6 (1.96)		12/20
ADDITIONAL CONTROL				
PNMHI	=1 if attending PNMHI School, 0 otherwise		19.6%	0/1
Paklihawa	=1 if attending Paklihawa School, 0 otherwise		31.7%	0/1
Purkot	=1 if attending school in Purkot, 0 otherwise (Base Category)		48.8%	0/1
HYGIENE PRODUCT USE				
Old Clothes	=1 if hygiene product currently using is old clothes, 0 otherwise		22.8%	0/1
Reusable	=1 if female currently using reusable hygiene product, 0 otherwise		10.3%	0/1
Disposable	=1 if female currently using disposable hygiene product, 0 otherwise		66.9%	0/1
CASTE				
Brahman Chhetri	=1 if female belongs to Brahman-Chhetri caste, 0 otherwise (Base)		47%	0/1
Dalit	=1 if female belongs to Dalit caste, 0 otherwise		11.4%	0/1
Madhesi	=1 if female belongs to Madhesi caste, 0 otherwise		16.4%	0/1
Other	=1 if female belongs to other castes, 0 otherwise		23.1%	0/1
Wealth Index	Sum of binary (1/0) self-reported yes answers to self/family owning land & cement home	1.38 (0.568)		0/2
Married	=1 if the female is married, 0 otherwise		3.6	0/1

**Table 2:** Single equation estimation of menstruation-related emotional stress & school absence ^1^.

	(1)	(2)	(3)
Variables	Emotional Stress ^2^ (Probit)	Missed School ^3^ (Probit)	Days Missed ^4^ (Oprobit)
Community Environment	0.116		
	(0.070)		
Family Environment	0.128		
	(0.075)		
School Environment	−0.299 **		
	(0.095)		
Emotional Stress		0.580 ***	0.341 *
		(0.170)	(0.161)
Age	1.096	−0.997	−0.958
	(0.614)	(0.619)	(0.557)
Age Sq.	−0.035	0.031	0.028
	(0.019)	(0.019)	(0.017)
Constant/Cut Point 1	−8.7	7.055	−7.132
	(5.042)	(5.052)	(4.651)
Cut Point 2			−5.924
			(4.659)
Fixed Effects ^5^	Yes	Yes	Yes
Caste ^6^	Yes	Yes	No
N	281	281	281
ln(L)	−180	−159	−192
χ^2^	29	32.9	25.5
AIC	381	336.4	398.3
BIC	421	369.2	423.7

Robust standard errors in parentheses; *** *p* < 0.01, ** *p* < 0.05, * *p* < 0.1. ^1^ Each model based on lowest AIC from estimation fitting for respective equation (see Tables S1–S3 in Supplementary Materials); ^2^ Dependent variable is binary indicator of whether girl feels sad/lonely during menstruation; ^3^ Dependent variable is binary indicator of whether girl misses school due to menstruation; ^4^ Dependent variable is ordinal indication of # missed days of school (1 = None, 2 = 1–2 days, 3 = 3+ days); ^5^ School-level fixed effects (Purkot as base); ^6^ Brahman-Chhetri (highest caste) as base.

**Table 3:** Bivariate probit estimation of menstruation-related emotional stress & school absence ^1^.

Explanatory Variables	MODEL 1		MODEL 2	
	Emotional Stress	Missing School	Emotional Stress	Missing School
Community Environment	0.158 **		0.146 *	
	(0.056)		(0.057)	
Family Environment	0.160 **		0.148 *	
	(0.061)		(0.058)	
School Environment	−0.221 **		−0.252 **	
	(0.083)		(0.083)	
Emotional Stress		1.737 ***		1.818 ***
		(0.183)		(0.120)
Age	1.191 *	−1.017	1.372 *	−0.957
	(0.600)	(0.568)	(0.606)	(0.571)
Age Sq.	−0.038 *	0.032	−0.044 *	0.03
	(0.019)	(0.018)	(0.019)	(0.018)
Constant	−9.463	6.651	−10.697 *	6.092
	(4.868)	(4.559)	(4.892)	(4.582)
Rho (ρ)	−0.917 ***		−1 ***	
	(0.176)		(2.20 × 10^−11^)	
Fixed Effects ^1^	Yes	Yes	Yes	Yes
Caste ^2^	No	No	Yes	Yes
N	281		281	
ln(L)	−341		−333	
AIC	713		706.6	

Robust standard errors in parentheses; *** *p* < 0.01, ** *p* < 0.05, * *p* < 0.1; ^1^ School-level fixed effects (Purkot as base); ^2^ Brahman-Chhetri (highest) as base.

**Table 4:** Marginal effects of bivariate probit estimation of menstruation-related emotional stress & school absence.

	MODEL 1		MODEL 2	
	Emotional Stress	Missing School ^1^	Emotional Stress	Missing School ^1^
Community Environment	0.0586 **		0.053 **	
	(0.022)		(0.020)	
Family Environment	0.0592 **		0.0537 *	
	(0.022)		(0.020)	
School Environment	−0.0819 **		−0.0916 ***	
	(0.030)		(0.0287)	
Emotional Stress		0.474 ***		0.489 ***
		(0.026)		(0.013)
Fixed Effects ^2^	Yes	Yes	Yes	Yes
Caste ^3^	No	No	Yes	Yes

Delta Method standard errors in parentheses; *** *p* < 0.01, ** *p* < 0.05, * *p* < 0.1; ^1^ Dependent variable is binary indicator (1/0) if missing school during menstruation; ^2^ School-level fixed effects (Purkot as base); ^3^ Brahman-Chhetri (highest caste) as base.

**Table 5:** Conditioned mixed process (CMP) estimation of menstruation-related emotional stress & school absence.

Explanatory Variables	MODEL 1		MODEL 2	
	Emotional Stress	Days Missed ^1^	Emotional Stress	Days Missed ^1^
Community Environment	0.085		0.08	
	(0.069)		(0.070)	
Family Environment	0.153 *		0.152 *	
	(0.061)		(0.062)	
School Environment	−0.206 **		−0.226 **	
	(0.069)		(0.074)	
Emotional Stress		1.630 ***		1.671 ***
		(0.131)		(0.142)
Age	1.274 *	−0.938	1.325 *	−1.001
	(0.585)	(0.567)	(0.597)	(0.585)
Age Sq.	−0.040 *	0.028	−0.042 *	0.031
	(0.018)	(0.017)	(0.018)	(0.018)
Constant/Cut 1	−10.157 *	−6.195	−10.488 *	−6.537
	(4.818)	(4.681)	(4.898)	(4.802)
Cut 2		−5.32		−5.636
		(4.670)		(4.789)
Rho (ρ)	−0.905 ***		−0.899 ***	
	(0.064)		(0.073)	
Fixed Effects ^2^	Yes	Yes	Yes	Yes
Caste ^3^	No	No	Yes	Yes
N	281		281	
ln(L)	−369.56		−364.24	
AIC	771.12		772.49	

Robust standard errors in parentheses; *** *p* < 0.01, ** *p* < 0.05, * *p* < 0.1; ^1^ Dependent variable is ordinal indication of # missed days of school (1 = None, 2 = 1–2 days, 3 = 3+ days); ^2^ School-level fixed effects (Purkot as base);^3^ Brahman-Chhetri (highest caste) as base.

**Table 6:** Marginal effects of conditioned mixed process (CMP) estimation of menstruation-related emotional stress & school absence.

	MODEL 1			MODEL 2		
Effect on No. Days Miss School ^1^	0 Days	1–2 Days	3+ Days	0 Days	1–2 Days	3+ Days
Emotional Stress	−0.454 ***	0.142 ***	0.312 ***	−0.465***	0.151 ***	0.314 ***
	(0.021)	(0.039)	(0.054)	(0.024)	(0.043)	(0.061)
Effects on Emotional Stress						
School Environment	−0.0771 **			−0.0836 ***		
	(0.025)			(0.026)		
Family Environment	0.0574 *			0.0563 *		
	(0.022)			(0.023)		
Community Environment	0.0317			0.0297		
	(0.026)			(0.026)		
Fixed Effects ^2^	Yes	Yes	Yes	Yes	Yes	Yes
Caste ^3^	No	No	No	Yes	Yes	Yes

Delta Method standard errors in parentheses; *** *p* < 0.01, ** *p* < 0.05, * *p* < 0.1; ^1^ Dependent variable is ordinal indication of # missed days of school (1 = None, 2 = 1–2 days, 3 = 3+ days); ^2^ School-level fixed effects (Purkot as base); ^3^ Brahman-Chhetri (highest caste) as base.

## Data Availability

The datasets used and/or analyzed during the current study are available from the corresponding author on reasonable request.

## Supplementary Materials

The following are available online at https://www.mdpi.com/article/10.3390/ijerph18178894/s1, Table S1: Model Selection of Equation (1)—Environmental Impacts on Emotional Health (Binary Outcome), Table S2: Model Selection of Equation (2), Specification A—Emotional Stress Impact on Missing School (Binary Outcome), Table S3: Model Selection of Equation (2), Specification B—Emotional Stress Impact on Days of Missed School (Ordinal Outcome), Table S4: Key Survey Questions.
