# Supplementary material for: The Stressor in Adolescence of Menstruation: Coping Strategies, Emotional Stress & Impacts on School Absences among Young Women in Nepal
Source: Int J Environ Res Public Health. 2021 Aug 24;18(17):8894. doi: 10.3390/ijerph18178894 (PMC8431475; doi:10.3390/ijerph18178894)
Supplement: Supplementary file 1 [file ijerph-18-08894-s001.zip › ijerph-1225188-supplementary.pdf]

Table S1: Model Selection of Equation 1—Environmental Impacts on Emotional Health (Binary Outcome)

VARIABLES	(1)	(2)	(3)	(4)	(5)	(6)	(7)
Community Cultural	0.108	0.135	0.116	0.137*	0.152*	0.114	0.131
Environ.	(0.067)	(0.069)	(0.070)	(0.069)	(0.070)	(0.070)	(0.071)
Family Cultural	0.112	0.11	0.128	0.104	0.104	0.12	0.123
Environ.	(0.076)	(0.074)	(0.075)	(0.077)	(0.076)	(0.078)	(0.077)
School Support	−0.123 *	−0.254**	−0.299 **	−0.188 **	−0.273 **	−0.206 **	−0.312 **
Environ.	(0.061)	(0.093)	(0.095)	(0.068)	(0.094)	(0.071)	(0.096)
Age	0.837	1.083	1.096	0.828	0.992	0.829	1.074
	(0.574)	(0.614)	(0.614)	(0.587)	(0.624)	(0.608)	(0.622)
Age Sq.	−0.025	−0.034	−0.035	−0.024	−0.031	−0.025	−0.034
	(0.018)	(0.019)	(0.019)	(0.018)	(0.019)	(0.019)	(0.019)
Constant	−7.006	−8.665	−8.7	−7.543	−8.402	−7.197	−8.795
	(4.639)	(5.059)	(5.042)	(4.763)	(5.121)	(4.961)	(5.093)
Fixed Effects[1]	No	Yes	Yes	No	Yes	No	Yes
Caste[2]	No	No	Yes	No	No	Yes	Yes
Control[3]	No	No	No	Yes	Yes	Yes	Yes
N	281	281	281	281	281	281	281
ln (L)	−186	−183	−180	−182	−181	−179	−178
χ^2	17.6	22.8	29	23.9	27.9	31.4	32.8
AIC	383.1	382.1	381	384.1	385.1	384.2	385
BIC	404.9	411.3	421	420.4	428.7	431.5	439.6

Robust standard errors in parentheses; *** $p < 0.01$, ** $p < 0.05$, * $p < 0.1$; [1] School-level Fixed Effects (Purkot as Base Category); [2] Brahman-Chhetri (highest caste) as Base Category; [3] Includes dummies for current hygiene product use type (old rags/cloths as base category), marriage dummy, wealth index indicator for cement home and owning land

Table S2: Model Selection of Equation 2, Specification A—Emotional Stress Impact on Missing School (Binary Outcome)

VARIABLES	(1)	(2)	(3)	(4)	(5)	(6)	(7)
Emotional Stress	0.490**	0.548***	0.580***	0.515**	0.577***	0.525**	0.598***
	(0.161)	(0.166)	(0.170)	(0.163)	(0.168)	(0.167)	(0.172)
Age	−1.526**	−0.976	−0.997	−1.406*	−0.905	−1.526*	−0.974
	(0.553)	(0.596)	(0.619)	(0.558)	(0.603)	(0.596)	(0.631)
Age Sq.	0.049**	0.03	0.031	0.045**	0.027	0.049**	0.03
	(0.017)	(0.018)	(0.019)	(0.017)	(0.019)	(0.018)	(0.020)
Constant	11.068*	6.972	7.055	9.547*	6.48	10.532*	6.852
	(4.426)	(4.856)	(5.052)	(4.511)	(4.861)	(4.824)	(5.089)
Fixed Effects[1]	No	Yes	Yes	No	Yes	No	Yes
Caste[2]	No	No	Yes	No	No	Yes	Yes
Control[3]	No	No	No	Yes	Yes	Yes	Yes
N	281	281	281	281	281	281	281
ln (L)	−169	−163	−159	−165	−160	−163	−156
χ^2	16.1	28.8	32.9	25.1	36.6	29.2	41
AIC	346	337.1	336.4	345.6	339.1	347.3	338.5
BIC	360.5	358.9	369.2	374.7	375.4	387.4	385.8

Robust standard errors in parentheses; *** $p < 0.01$, ** $p < 0.05$, * $p < 0.1$; [1] School-level Fixed Effects (Purkot as Base Category); [2] Brahman-Chhetri (highest caste) as Base Category; [3] Includes dummies for current hygiene product use type (old rags/cloths as base category), marriage dummy, wealth index indicator for cement home and owning land.

Table S3: Model Selection of Equation 2, Specification B—Emotional Stress Impact on Days of Missed School (Ordinal Outcome)

VARIABLES	(1)	(2)	(3)	(4)	(5)	(6)	(7)
Emotional Stress	0.305	0.341*	0.400*	0.330*	0.381*	0.373*	0.432**
	(0.156)	(0.161)	(0.166)	(0.157)	(0.162)	(0.163)	(0.166)
Age	−1.386**	−0.958	−1.037	−1.318*	−0.912	−1.440*	−1.052
	(0.523)	(0.557)	(0.579)	(0.540)	(0.570)	(0.559)	(0.593)
Age Sq.	0.043**	0.028	0.031	0.041*	0.026	0.045**	0.031
	(0.016)	(0.017)	(0.017)	(0.016)	(0.017)	(0.017)	(0.018)
Cut Point 1	−10.141*	−7.132	−7.587	−9.184*	−6.93	−9.895*	−7.693
	(4.283)	(4.651)	(4.829)	(4.463)	(4.723)	(4.596)	(4.899)
Cut Point 2	−8.961*	−5.924	−6.36	−7.972	−5.689	−8.67	−6.434
	(4.282)	(4.659)	(4.835)	(4.464)	(4.728)	(4.598)	(4.903)
Fixed Effects[1]	No	Yes	Yes	No	Yes	No	Yes
Caste[2]	No	No	Yes	No	No	Yes	Yes
Control[3]	No	No	No	Yes	Yes	Yes	Yes
N	281	281	281	281	281	281	281
ln (L)	−198	−192	−189	−194	−188	−191	−186
χ^2	11.3	25.5	30.6	21.2	38.1	24	43.3
AIC	405.2	398.3	398.9	405.1	398.5	407	399.5
BIC	423.4	423.7	435.3	437.9	438.5	450.6	450.4

Robust standard errors in parentheses; *** $p < 0.01$, ** $p < 0.05$, * $p < 0.1$; [1] School-level Fixed Effects (Purkot as Base Category); [2] Brahman-Chhetri (highest caste) as Base Category; [3] Includes dummies for current hygiene product use type (old rags/cloths as base category), marriage dummy, wealth index indicator for cement home and owning land

Table S4. Key Survey Questions

**Outcomes**		**Options**
Em_i^*	Do you feel lonely and sad during your menstruation cycle?	Yes
		No
Sch_i^* (Spec. A)	Have you missed your school due to your menstruation?	Yes
		No
Sch_i^* (Spec. B)	How many days in the last month have you missed school due to your menstruation?	1–2 days
		3–5 days
		Others
**Cultural Environment Questions (*CommEnvi & FamEnvi*)**		**Options**
1.	Are you allowed to enter prayer room during your menstruation cycle?	Yes
		No
2.	Do you stay in a separate house during menstruation?	Yes

		No
3.	Have you participated in cultural functions during menstruation?	Yes
		No
4.	Are you allowed in the kitchen during your menstruation cycle?	Yes
		No
5.	Do you live life during menstruation as if it were a normal day?	Yes
		No
6.	Are you allowed to meet with your family and friends like every other normal day during your menstruation cycle?	Yes
		No
**School Environment Questions (*SchEnv$_i$*)**		**Options**
1.	Do you get sanitation supplies in school in case you need it in emergency?	Yes
		No
2.	Do you have a separate toilet for girls where you can change your menstruation materials?	Yes
		No
3.	Do you get soap/ liquid lotion to wash your hand after you change your menstruation material in school?	Yes
		No
4.	Do you have a proper disposable bin where you can dispose your menstruation materials in school?	Yes
		No
		No
5.	Do you get to learn within school curriculum about recommended hygiene practices that you should follow and some guidelines about menstruation cycle?	Yes
		No
